# Barriers and facilitating factors in the prevention of diabetes type II and gestational diabetes in vulnerable groups: protocol for a scoping review

**DOI:** 10.1186/s13643-018-0919-y

**Published:** 2018-12-23

**Authors:** Jessica Breuing, Dawid Pieper, Annika Lena Neuhaus, Simone Heß, Lena Lütkemeier, Fabiola Haas, Mark Spiller, Christine Graf

**Affiliations:** 1 Institute for Research in Operative Medicine (IFOM), Faculty of Health, Department of Medicine, Witten/Herdecke University, Ostmerheimer Str. 200, Building 38, 51109 Cologne, Germany; 2 Institute of Movement and Neurosciences, German Sport University Cologne, Am Sportpark Müngersdorf 6, 50933 Cologne, Germany

**Keywords:** Diabetes mellitus type 2, Prevention, Vulnerable groups, Barriers and facilitating factors

## Abstract

**Background:**

There is a significant worldwide increase in type 2 diabetes mellitus and gestational diabetes (T2DM/GDM) linked to a range of associated comorbidities and rising healthcare costs. It has been shown that an increase in physical activity, healthy nutrition, and weight loss may prevent or delay T2DM/GDM manifestation. Despite this, it remains a key challenge to reach various populations, in particular so-called vulnerable groups, mostly with a migration background and/or low socio-economic status.

**Methods/design:**

We will conduct a scoping review to identify barriers and facilitating factors in the prevention of T2DM/GDM in vulnerable groups. An electronic literature search will be performed in MEDLINE, EMBASE, PsycINFO, PSYNDEX, Social Science Citation Index, and CINAHL. Two reviewers will independently select studies for inclusion. Extracted data will be charted, categorized, and summarized.

**Discussion:**

The results will be used to inform the National education and communication strategy on diabetes mellitus in Germany. In particular, the results will be discussed in focus groups of experts to develop recommendations for developing preventive measures targeting vulnerable groups.

**Systematic review registration:**

PROSPERO does not register scoping reviews.

**Electronic supplementary material:**

The online version of this article (10.1186/s13643-018-0919-y) contains supplementary material, which is available to authorized users.

## Background

Worldwide, the prevalence of type 2 (T2DM) and gestational diabetes (GDM) has risen and so have the associated health consequences and healthcare costs [1]. Evidence shows that the prevalence of T2DM/GDM is higher in obese, overweight and physically inactive individuals [2, 3]. An increase in physical activity, healthy nutrition, and weight loss may prevent or delay T2DM/GDM manifestation [4]. However, it is challenging to reach certain populations, in particular so-called vulnerable groups that include individuals with a migration background and/or low socio-economic status. Those patients are disproportionally affected by T2DM/GDM and diabetes-related complications [5, 6]. Language, cultural perception, shame, and lower health literacy often play an important role in non-participation [7]. Research suggests that behavioral change is possible, but this change generally requires comprehensive approaches tailored to specific settings and target groups [8].Therefore, tailored T2DM/GDM interventions should be informed by evidence of barriers and facilitating factors. Our target audiences are primary care providers (e.g., general practitioners, nutritionists, and midwifes) as well as diabetologists and public health experts active in diabetes prevention.

## Research objective

The aim of this scoping review is to identify and describe barriers and facilitating factors in the prevention of T2DM and GDM in vulnerable groups.

## Methods

This project was commissioned by Federal Centre for Health Education in Germany as part of the “National education and communication strategy on diabetes mellitus”. This is one of two scoping reviews, both of which will use the same search strategy and are similar in their methodology.

### Protocol

This protocol was prepared according to PRISMA-P [9]. The scoping review will be conducted following the Arksey and O’Malleys framework [10] and The Joanna Briggs Institute Reviewers’ Manual 2015 [11] .

### Eligibility criteria

Inclusion criteria:

- Vulnerable patients with, or at risk of T2DM or GDM
- Studies present barriers and facilitating factors for implementing a preventative or health-promoting intervention
- WHO mortality stratum A countries
- Publication date ≥ 2008

Exclusion criteria:

- Native people, children, or people with mental disorders
- No full texts are available

Eligibility criteria are additionally shown in the Population, Concept, Context (PCC) mnemonic in Table 1. We will include studies presenting barriers and facilitating factors for taking a preventative or health-promoting intervention for vulnerable patients with or at risk of T2DM and GDM. All types of studies will be included only if published from January 2008 onwards, for the following reason. Barriers and facilitators are affected by external factors such as accessibility of care and information. We assume that there has been a change in accessibility due to the volume of digital and virtual goods, services, and processes in healthcare over the past 10 years. As a result, the barriers and facilitators might have changed, so that there would be a lack of comparability if we chose a longer period.

**Table 1:** PCC (Population, Concept, Context)

P	Diabetes mellitus type II or gestation diabetes	Type 2 diabetes mellitus Gestational diabetes mellitus People at risk of developing diabetes mellitus or gestational diabetes mellitus
	Vulnerable patients/groups	Elderly, older people, seniors > 65 years Disabled people People in need of care, residents of a nursing home Unemployed people Refugees/migrants as well as ethnic groups (e.g., African Americans or Hispanics) Homeless people Drug/substance abusers (excluding nicotine abuse/smoking) Low socio-economic status
C	Prevention	Primary/secondary prevention
	Communication strategies	Communication strategies/access routes, e.g., digital/social media, TV/radio, print media, group sessions, health campaign
C	> 2008; WHO stratum A	
Other	All types of studies; all languages; available in full text version	

The PPC (Population, Concept, Context) mnemonic illustrates the eligibility criteria for the scoping review. Additionally to the classic PPC mnemonic, there are other criteria regarding study types, languages, and the availability of the full-text version

No language restrictions will be applied. All full texts published in languages other than English or German will be translated by an external agency. We will only include studies performed in countries within the low mortality stratum (A) according to the World Health Organization [12]. By doing so, we will ensure that our findings will be applicable throughout western industrialized countries. We define vulnerable groups according to Lewis et al. [13] (Table 1: PCC), but exclude native people, children, and people with mental disorders.

### Information sources

The following electronic databases will be searched: MEDLINE, EMBASE, PsycINFO, PSYNDEX, Social Science Citation Index, and CINAHL. Grey literature will be searched in greylit.org and through the homepages of the WHO and international healthcare or public health departments (e.g., Department of Health & Social Care, UK; Agency for Healthcare Research and Quality (AHRQ); US Preventive Services Task Force). We will search manually for additional studies by cross-checking the reference lists of all included studies.

### Search

The search strategy will be developed by the research team in collaboration with an experienced librarian and checked by a referee according to the Peer Review of Electronic Search Strategies (PRESS) guideline [14]. A draft of the PubMed search strategy is attached in the Additional file 1.

### Data management

The search results will be uploaded and managed using Microsoft Excel.

### Study selection

Two reviewers will independently screen titles and abstracts of search results and then assess selected full-text reports for eligibility. Any disagreement will be resolved by discussion and consensus or, if needed, by consultation with a third reviewer. The reasons for exclusion of full texts will be documented. A list of excluded studies will be provided. The corresponding authors of eligible articles will be contacted for clarification where necessary.

### Data extraction

A standardized extraction form will be developed for this review. The data extraction form will be piloted on a sample of five articles by the reviewers involved in the scoping review and will be assessed for completeness and applicability. Based on the pilot phase, the standardized data extraction form will be modified as required to ensure that all data necessary to address the research question are obtained. Data will be extracted by one reviewer and checked by another. Disagreements will be resolved through discussion and consensus.

### Data items

The preliminary data-extraction categories will be derived from our overarching research question. The following data will be collected:

- Study characteristics (e.g., country, setting, publication date, number of participants, target disease, study design/method)
- Patients’ characteristics (e.g., age, gender, affiliation to vulnerable group)
- Inclusion/exclusion criteria
- Barriers
- Facilitating factors

### Risk of bias

As this is a scoping review, there will be no risk of bias assessment. This is consistent with relevant guidance [10].

### Data analysis

We will use Arksey and O’Malley’s methods of reporting [10] and provide a descriptive analysis of the extent, nature, and distribution of the studies included in the review as well as a narrative, thematic summary of the data collected. This will be achieved by summarizing the literature according to the types of vulnerable groups, comparators, barriers and facilitating factors, and study outcomes. We aim to map the research landscape in this area. This will be supported by some form of visual representation of the data to map the extent, range, and nature of research in this area. Data will be charted, categorized, and summarized. We will report quantitative (e.g., frequency) and qualitative results. Furthermore, we will seek to explore similarities and differences, both within and between studies, to identify patterns and themes and postulate explanations for findings. In particular, we will focus on barriers and facilitating factors for participating in preventative intervention for vulnerable patients with, or at risk of, T2DM or GDM. By doing so, we will also consider the robustness of the included studies itself by reporting the overall strength and confidence of the findings. If possible, we will stratify our results by types of vulnerable groups.

## Discussion

The main aim of this review is to identify barriers and facilitating factors in the prevention of T2DM and GDM in vulnerable groups. The results will be used to inform the National education and communication strategy on diabetes mellitus in Germany. In particular, the results will be discussed in focus groups of experts to develop recommendations for developing preventive measures targeting vulnerable groups.

As this review is part of the “National awareness and prevention strategy on diabetes in Germany” conducted by the Federal Centre for Health Education and the Federal Ministry of Health, there is a narrow time frame for completing the report, and therefore, we have to limit the publication date. However, there might be too many differences in barriers and facilitating factors due to digitalization. The results of this review will be used to make appropriate recommendations on the development of preventative measures targeting vulnerable groups which could be used in different German healthcare settings. Another strength of this study will be the systematic search for all published literature on that topic. As this review is part of the overall project commissioned by the Federal Centre for Health Education, it will have national coverage in improving health education.

## Additional file

Additional file 1: Search strategy (MEDLINE). (DOCX 14 kb)

## Abbreviations

GDM: Gestational diabetes
PPC: Population, Concept, Context
T2DM: Type 2 diabetes mellitus
WHO: World Health Organization
