# Correction to: Barriers and facilitating factors in the prevention of diabetes type II and gestational diabetes in vulnerable groups: protocol for a scoping review

**DOI:** 10.1186/s13643-019-0944-5

**Published:** 2019-01-22

**Authors:** Jessica Breuing, Dawid Pieper, Annika Lena Neuhaus, Simone Heß, Lena Lütkemeier, Fabiola Haas, Mark Spiller, Christine Graf

**Affiliations:** 1 Institute for Research in Operative Medicine (IFOM), Faculty of Health, Department of Medicine, Witten/Herdecke University, Ostmerheimer Str. 200, Building 38, 51109 Cologne, Germany; 2 Institute of Movement and Neurosciences, German Sport University Cologne, Am Sportpark Müngersdorf 6, 50933 Cologne, Germany

**Correction to: Syst Rev**

**https://doi.org/10.1186/s13643-018-0919-y**

Following publication of the original article [1], the authors reported an error in Table 1 as the wrong table was used. The inclusion criteria of the scoping review has been corrected in Table 1 here. Please see below. The authors would like to apologize for this error.

**Table 1:** PCC (Population, Concept, Context)

P	Diabetes mellitus type 2 or gestation diabetes	Type 2 diabetes mellitus
		Gestational diabetes mellitus
		People at risk of developing diabetes mellitus or gestational diabetes mellitus
	Vulnerable patients/−groups	Elderly, older people, seniors > 65 years
		Disabled people
		People in need of care, residents of a nursing home
		Unemployed people
		Refugees/migrants as well as ethnic groups (e.g. African Americans or Hispanics)
		Homeless people
		Drug/substance abusers (excluding nicotine abuse/smoking)
		Low socio-economic status
C	Prevention	Primary/ secondary/ tertiary prevention
	Barriers and facilitating factors	Definition of barriers and motivating aspects e.g. language, costs, religion, ethnic background, low income, social and health support
		Solutions to exploit barriers and support solutions e.g. materials and manpower, use of media, insurance
C	> 2008; WHO stratum A	
Other	all types of studies; all languages; available in full text version	

The PPC (Population, Concept, Context) mnemonic illustrates the eligibility criteria for the scoping review. Additionally to the classic PPC mnemonic there are other criteria regarding study types, languages and the availability of the full text version

## References

[1] Breuing J, Pieper D, Neuhaus AL, Heß S, Lütkemeier L, Haas F, Spiller M, Graf C (2018). Barriers and facilitating factors in the prevention of diabetes type II and gestational diabetes in vulnerable groups: protocol for a scoping review. Syst Rev.

