# Supplementary material for: Barriers and facilitating factors in the prevention of diabetes type II and gestational diabetes in vulnerable groups: protocol for a scoping review
Source: Syst Rev. 2018 Dec 23;7:245. doi: 10.1186/s13643-018-0919-y (PMC6304233; doi:10.1186/s13643-018-0919-y)
Supplement: Supplementary file 1 — Search strategy (MEDLINE). (DOCX 14 kb) [file 13643_2018_919_MOESM1_ESM.docx]

Search strategy (Medline)

"Diabetes, Gestational"[Mesh] OR "Diabetes Mellitus, Type 2"[Mesh] OR gestational diabetes[tiab] OR diabetes mellitus, gestational[tiab] OR pregnancy-induced diabetes[tiab] OR type 2 diabetes[tiab] OR “diabetes mellitus type II”[tiab] OR type 2 diabetes mellitus[tiab] OR “diabetes type 2”[tiab]

AND

("Ethnic Groups"[Mesh] OR "Minority Groups"[Mesh] OR "Poverty Areas"[Mesh] OR "Vulnerable Populations"[Mesh] OR "Health Status Disparities"[Mesh] OR "Cultural Diversity"[Mesh] OR "Socioeconomic Factors"[Mesh] OR "Aged"[Mesh] OR "Substance-Related Disorders"[Mesh] OR "Malnutrition"[Mesh] OR "Disabled Persons"[Mesh] OR "Educational Status"[Mesh] OR "Emigrants and Immigrants"[Mesh] OR "Homeless Persons"[Mesh] OR "Minors"[Mesh] OR "Transients and Migrants"[Mesh] OR "Refugees"[Mesh] OR "Unemployment"[Mesh] OR "Mental Disorders"[Mesh] OR ethnic group*[tiab] OR ethnic population*[tiab] OR minority[tiab] OR minorities[tiab] OR ethnic minorit*[tiab] OR poverty[tiab] OR destitution[tiab] OR poor housing[tiab] OR addiction[tiab] OR drug abuse[tiab] OR malnutrition[tiab] OR malnourished[tiab] OR vulnerable population*[tiab] OR vulnerable group*[tiab] OR socioeconomic factor*[tiab] OR socioeconomic aspect*[tiab] OR low socioeconomic status[tiab] OR deprived[tiab] OR health status[tiab] OR aged[tiab] OR elderly[tiab] OR elders[tiab] OR minors[tiab] OR disabled[tiab] OR disability[tiab] OR level of education[tiab] OR education level[tiab] OR low level of education[tiab] OR mental disorder[tiab] OR need for care[tiab] OR need of care[tiab] OR care dependency[tiab] OR unemployment[tiab] OR ethnic disparity[tiab] OR ethnic disparities[tiab] OR migrant[tiab] OR migrants[tiab] OR immigrant[tiab] OR immigrants[tiab] OR asylum[tiab] OR refugees[tiab] OR cultural diversity[tiab] OR multicultural aspect*[tiab] OR multicultural factor*[tiab] OR religion[tiab] OR homeless*[tiab])

AND

(“Primary Prevention"[Mesh] OR "Secondary Prevention"[Mesh] OR "Tertiary Prevention"[Mesh] OR "Preventive Health Services"[Mesh] OR "Mass Screening"[Mesh] OR "Health Promotion"[Mesh] OR "Health Education"[Mesh] OR "Patient Education as Topic"[Mesh] OR "Health Literacy"[Mesh] OR "Health Services for Persons with Disabilities"[Mesh] OR "Health Services for the Aged"[Mesh] OR "Health Services, Indigenous"[Mesh] OR "Culturally Competent Care"[Mesh] OR prevention[tiab] OR prevent[tiab] OR preventing[tiab] OR health service*[tiab] OR screening[tiab] OR health promotion[tiab] OR health education[tiab] OR patient education[tiab] OR health literacy[tiab] OR health care[tiab])

AND

("Health Communication"[Mesh] OR "Reminder Systems"[Mesh] OR "Counseling"[Mesh] OR "Communications Media"[Mesh] OR "Motivation"[Mesh] OR "Information Dissemination"[Mesh] OR "Consumer Health Information"[Mesh] OR "Pamphlets"[Mesh] OR "Information Literacy"[Mesh] OR "Teaching Materials"[Mesh] OR intervention[tiab] OR interventions[tiab] OR health communication[tiab] OR communication media[tiab] OR reminder system*[tiab] OR counseling[tiab] OR counselling[tiab] OR health information[tiab] OR information dissemination[tiab] OR information literacy[tiab] OR teaching material*[tiab] OR pamphlet[tiab] OR pamphlets[tiab] OR booklet[tiab] OR booklets[tiab] OR leaflet[tiab] OR leaflets[tiab] OR flyer[tiab] OR flyers[tiab] OR poster[tiab] OR posters[tiab] OR brochure[tiab] OR brochures[tiab] OR access[tiab] OR communication strategy[tiab] OR communication strategies[tiab] OR strategy[tiab] OR strategies[tiab] OR audio*[tiab] OR video[tiab] OR videos[tiab] OR dvd*[tiab] OR compact disc*[tiab] OR cd[tiab] OR cds[tiab] OR "Multimedia"[Mesh] OR multimedia[tiab] OR multi-media[tiab] OR media campaign[tiab] OR "Telecommunications"[Mesh] OR "Internet"[Mesh] OR internet[tiab] OR web[tiab] OR website*[tiab] OR online[tiab] OR electronic mail*[tiab] OR email*[tiab] OR mail*[tiab] OR "Blogging"[Mesh] OR blog*[tiab] OR weblog*[tiab] OR podcast*[tiab] OR portal*[tiab] OR computer program*[tiab] OR computer mediated[tiab] OR computer based[tiab] OR computer assisted[tiab] OR "Correspondence as Topic"[Mesh] OR telephon*[tiab] OR phone[tiab] OR phones[tiab] OR text messag*[tiab] OR sms[tiab] OR facilitator[tiab] OR facilitators[tiab] OR facilitate[tiab] OR motivation[tiab] OR motivators[tiab] OR motivational strategy[tiab] OR motivational strategies[tiab] OR enablers[tiab] OR promotional[tiab] OR beneficial[tiab] OR helpful[tiab] OR fostering[tiab] OR advantageous[tiab] OR barrier[tiab] OR barriers[tiab] OR barricade[tiab] OR impeding[tiab] OR hindering[tiab])

NOT ("Comment" [Publication Type] OR "Letter" [Publication Type] OR "Editorial" [Publication Type])
